# Height-Specific Serum Cholesterol Levels in Pubertal Children: Data From Population-Based Japanese School Screening

**DOI:** 10.2188/jea.JE20100108

**Published:** 2011-03-05

**Authors:** Yuki Fujita, Katsuyasu Kouda, Harunobu Nakamura, Nobuhiro Nishio, Hiroichi Takeuchi, Masayuki Iki

**Affiliations:** 1 Department of Public Health, Kinki University Faculty of Medicine, Osaka-Sayama, Osaka, Japan; 2 Department of Health Promotion and Education, Graduate School of Human Development and Environment, Kobe University, Kobe, Japan; 3 Department of Public Health, Wakayama Medical University, Wakayama, Japan; 4 Hamamatsu University School of Medicine, Hamamatsu, Japan

**Keywords:** child, diagnosis, growth, cholesterol

## Abstract

**Background:**

Inverse associations between height and serum lipid levels in pubertal children have been reported. To develop criteria for normal serum lipid levels stratified by height in pubertal children, we examined height-specific cholesterol levels, using data from a population-based school screening.

**Methods:**

Serum levels of total cholesterol (TC) and high-density lipoprotein cholesterol (HDL-C) were investigated in 10 151 children (98.9% of the target population) aged 10 and 14 years who attended public schools in Iwata City from 2002 through 2007.

**Results:**

The 95th percentiles of TC in the lowest and highest quintiles of height were 221 and 219 mg/dL, respectively, in 10-year-old boys, 215 and 203 mg/dL in 14-year-old boys, 220 and 204 mg/dL in 10-year-old girls, and 226 and 214 mg/dL in 14-year-old girls. The fifth percentiles of HDL-C in the lowest and highest quintiles of height were 45 and 43 mg/dL in 10-year-old boys, 43 and 40 mg/dL in 14-year-old boys, 46 and 42 mg/dL in 10-year-old girls, and 47 and 44 mg/dL in 14-year-old girls.

**Conclusions:**

This study provided height-specific levels of serum lipids in 10-year-old and 14-year-old children. Height should be considered when evaluating cholesterol levels in pubertal children.

## INTRODUCTION

Adverse levels of serum lipoprotein cholesterols are known risk factors for coronary artery disease and atherosclerosis.^1^ In a given individual, serum lipid and lipoprotein levels tend to be consistent from childhood into young adulthood.^2^ In addition, atherosclerosis begins very early in life, and elevated lipoprotein levels in childhood are associated with coronary atherosclerosis in adulthood.^2^^–^^4^ These findings highlight the necessity of measuring serum cholesterol early in life, as well as the need for prevention and intervention aimed at developing healthy lifestyles.^2^ In the United States, a cholesterol lowering project was recommended for children and adolescents by the National Cholesterol Educational Program (NCEP).^5^ The NCEP aims to raise awareness and understanding of dyslipidemia as a risk factor for coronary artery disease.^5^ Numerous school-based screening and intervention programs have been conducted in pubertal children for the purpose of cardiovascular disease prevention.^6^^–^^10^ In Japan, cholesterol screening of schoolchildren in fourth to ninth grade has been conducted since the early 1990s,^11^^–^^13^ and criteria for normal cholesterol levels in children have been reported using a large nationwide dataset.^14^

At puberty, dynamic changes occur in serum lipid levels, which peak at age 9 to 10 years (fourth grade) and then decrease. In adolescence, serum lipids begin to increase again.^15^^–^^20^ In addition, height growth velocity is inversely associated with dynamic changes in serum lipids during puberty^21^^,^^22^: adolescents who experience a large increase in height tend to show a decrease in serum lipid levels, while those who experience a small increase in height tend to show an increase in serum lipid levels.^11^ However, the current criteria for normal cholesterol levels in pubertal children do not account for height growth. Therefore, it is necessary to develop criteria for normal serum lipid levels stratified by height in this population. The aim of this study was to calculate height-specific lipid levels in pubertal children, using data from population-based school screening in Iwata City, Japan.

## METHODS

### Study population

The city of Iwata is located in Shizuoka Prefecture, Japan, about 230 km from Tokyo. On 1 April 2005, Iwata merged with 4 other municipalities, and a new city of Iwata was created. In the present study, the subjects were students in fifth grade (age 10 years) and ninth grade (age 14 years) who attended any public school in the original area of Iwata from 2002 through 2007. There were no private schools in the original area of Iwata during that period, and almost all children residing in the area were enrolled in public schools. School screenings were conducted by a local government authority, the Iwata City Board of Education, from April through June each year. The characteristics of the annual health examination in Iwata children have been reported previously, and anthropometric indices and serum lipid levels of children in Iwata were similar to the reported average values for Japanese children.^23^ We analyzed data from 10 151 children (98.9% of the target population). The study was approved by the Ethics Committee of the Kinki University School of Medicine.

### Examination

Examinations were conducted annually at each school, as described previously.^23^ Height measurements were performed by nationally certified health education teachers (*yogo* teachers). The method used was in accordance with the Japanese School Health Law, and heights were measured to an accuracy of 0.1 cm. Blood samples were taken by nurses and medical technologists. Blood testing was conducted at a laboratory associated with the *Shizuokaken Yoboigakukyokai* (Shizuoka Prefecture Preventive Medicine Association, Shizuoka, Japan), and measurements were conducted at a laboratory of the organization. Total cholesterol (TC) levels were determined enzymatically (Pureauto S CHO-N, Daiichi Pure Chemical Co. Ltd, Tokyo, Japan) using a Hitachi 7350 automatic chemistry analyzer. High-density lipoprotein cholesterol (HDL-C) levels were determined using the direct method (Cholestest N HDL, Daiichi Pure Chemical Co. Ltd) with the same analyzer. The precision and accuracy of lipid determinations were controlled by internal quality control and by external quality assessment by the Japan Medical Association. Intra- and inter-laboratory coefficients of variation were less than 4%.

### Statistical analysis

Statistical calculations were performed using SAS software for Windows, version 9.1 (SAS Institute Japan Ltd, Tokyo, Japan). To assess the associations between height and various lipid levels in more detail, height values were divided into quintiles (20% of the study population in each quintile), and the 5th, 25th, 50th, 75th, and 95th percentiles of lipid levels were calculated according to height quintile. In accordance with a nationwide study of Japanese children in 19 prefectures and the US NCEP criteria for children and adolescents, the 75th through 95th percentile of TC was defined as borderline, and a value higher than the 95th percentile was defined as high. For HDL-C, the 5th through 25th percentile was defined as borderline, and a value below the 5th percentile was defined as low.^5^^,^^14^ Thus, the cut-off points for TC and non–HDL-C (TC minus HDL-C) in each quintile were defined as the 95th percentile, and those for HDL-C were defined as the 5th percentile. Height-specific cut-off points were calculated for subjects in each quintile, and non–height-specific cut-off points were calculated using data from all subjects. The McNemar test was used to compare height-specific and non–height-specific cut-off points.

## RESULTS

### Association between height and serum lipid levels

There were significant inverse associations of height with serum TC, HDL-C, and non–HDL-C, excluding serum HDL-C in girls at age 14 and serum non–HDL-C in boys at ages 10 and 14 (Table 1).

**Table 1.** Correlations between height and lipid variables

	Boys		Girls	
		
	Pearson’s correlation coefficient	*P* value	Pearson’s correlation coefficient	*P* value
TC				
Age 10	−0.05	<0.01	−0.20	<0.01
Age 14	−0.10	<0.01	−0.06	<0.01
HDL-C				
Age 10	−0.16	<0.01	−0.15	<0.01
Age 14	−0.13	<0.01	0.03	0.19
Non–HDL-C				
Age 10	0.03	0.19	−0.15	<0.01
Age 14	−0.04	0.06	−0.09	<0.01

TC, total cholesterol; HDL-C, high-density lipoprotein cholesterol.

### Height

The mean height difference between the lowest and highest quintiles was 17 cm in 10-year-old boys, 19 cm in 14-year-old boys, 19 cm in 10-year-old girls, and 15 cm in 14-year-old girls (Tables 2, 3, and 4).

**Table 2.** Mean height and distribution of height-specific serum TC concentration

	Height quintile (boys)						Height quintile (girls)					
		
	Total	1st	2nd	3rd	4th	5th	Total	1st	2nd	3rd	4th	5th
	10-year-olds											
Mean height (cm)		130	135	138	141	147		130	136	139	143	149
TC (mg/dL)												
50th percentile	170	172	172	173	168	166	170	178	172	172	167	162
75th percentile	189	190	193	189	189	185	188	195	189	190	183	181
95th percentile	221	221	223	220	223	219	217	220	221	223	212	204

	14-year-olds											
Mean height (cm)		155	162	165	169	174		149	154	156	159	164
TC (mg/dL)												
50th percentile	158	162	158	157	158	157	171	172	174	170	173	168
75th percentile	175	183	176	173	172	171	190	191	190	188	193	188
95th percentile	206	215	203	206	200	203	223	226	224	219	225	214

TC, total cholesterol.

**Table 3.** Mean height and distribution of height-specific serum HDL-C concentration

	Height quintile (boys)						Height quintile (girls)					
		
	Total	1st	2nd	3rd	4th	5th	Total	1st	2nd	3rd	4th	5th
	10-year-olds											
Mean height (cm)		130	135	138	141	147		130	136	139	143	149
HDL-C (mg/dL)												
50th percentile	64	66	65	65	62	60	62	65	64	62	61	59
25th percentile	56	58	57	56	55	52	54	56	56	55	53	52
5th percentile	45	45	45	45	44	43	44	46	47	43	43	42

	14-year-olds											
Mean height (cm)		155	162	165	169	174		149	154	156	159	164
HDL-C (mg/dL)												
50th percentile	59	62	60	60	58	57	63	62	63	63	64	63
25th percentile	52	54	53	51	51	51	56	56	56	55	56	56
5th percentile	43	43	43	43	42	40	46	47	48	44	46	44

HDL-C, high-density lipoprotein cholesterol.

**Table 4.** Mean height and distribution of height-specific serum non–HDL-C concentration

	Height quintile (boys)						Height quintile (girls)					
		
	Total	1st	2nd	3rd	4th	5th	Total	1st	2nd	3rd	4th	5th
	10-year-olds											
Mean height (cm)		130	135	138	141	147		130	136	139	143	149
Non–HDL-C (mg/dL)												
50th percentile	105	104	107	108	104	105	107	112	108	108	105	102
75th percentile	122	121	123	122	123	122	123	127	123	126	122	119
95th percentile	154	151	154	152	157	155	150	153	153	155	147	144

	14-year-olds											
Mean height (cm)		155	162	165	169	174		149	154	156	159	164
Non–HDL-C (mg/dL)												
50th percentile	98	100	98	96	99	95	106	108	107	105	108	102
75th percentile	112	114	112	112	112	112	124	128	126	123	125	120
95th percentile	141	143	141	143	137	142	151	158	155	150	151	145

HDL-C, high-density lipoprotein cholesterol.

### TC level

The 95th percentiles of TC level in the lowest and highest quintiles of height were 221 and 219 mg/dL, respectively, in 10-year-old boys, 215 and 203 mg/dL in 14-year-old boys, 220 and 204 mg/dL in 10-year-old girls, and 226 and 214 mg/dL in 14-year-old girls (Table 2). The difference in the TC cut-off point between the lowest and highest quintiles of height was 16 mg/dL in 10-year-old girls and 12 mg/dL in 14-year-old boys and girls.

### HDL-C level

The fifth percentiles of HDL-C level in the lowest and highest quintiles of height were 45 and 43 mg/dL, respectively, in 10-year-old boys, 43 and 40 mg/dL in 14-year-old boys, 46 and 42 mg/dL in 10-year-old girls, and 47 and 44 mg/dL in 14-year-old girls (Table 3). The difference in the HDL-C cut-off point between the lowest and highest quintiles of height was 2 and 4 mg/dL in 10-year-old boys and girls, respectively, and 3 mg/dL in 14-year-old boys and girls.

### Non–HDL-C level

The 95th percentiles of non–HDL-C level in the lowest and highest quintiles of height were 153 and 144 mg/dL, respectively, in 10-year-old girls and 158 and 145 mg/dL in 14-year-old girls (Table 4). The difference in the cut-off point for non–HDL-C between the lowest and highest quintiles of height was 9 mg/dL in 10-year-old girls and 13 mg/dL in 14-year-olds girls.

### Prevalence of dyslipidemia

Among both boys and girls, there were significant differences in the prevalence of dyslipidemia calculated using height-specific and non–height-specific cut-off points (Tables 5 and 6).

**Table 5.** Prevalence of boys with dyslipidemia by height-specific and non–height-specific cut-off points in each quintile

	Height quintile	Dyslipidemia, number (%)					
	
		Height-specific cut-off point	Non–height-specific cut-off point	Height-specific cut-off point	Non–height-specific cut-off point	Height-specific cut-off point	Non–height-specific cut-off point
		TC		HDL-C		Non–HDL-C	
Age 10	1st	28 (5.0)	28 (5.0)	29 (5.2)	29 (5.2)	29 (5.2)	23 (4.1)^b^
	2nd	27 (5.1)	29 (5.5)	27 (5.1)	27 (5.1)	28 (5.3)	28 (5.3)
	3rd	28 (5.2)	24 (4.5)	29 (5.4)	29 (5.4)	28 (5.2)	25 (4.7)
	4th	30 (5.5)	31 (5.7)	30 (5.5)	35 (6.4)	30 (5.5)	35 (6.4)
	5th	29 (5.4)	26 (4.8)	32 (5.9)	47 (8.7)^a^	27 (5.0)	29 (5.4)

Age 14	1st	28 (5.1)	46 (8.4)^a^	29 (5.3)	29 (5.3)	28 (5.1)	32 (5.9)
	2nd	29 (5.4)	22 (4.1)^b^	29 (5.4)	29 (5.4)	27 (5.0)	27 (5.0)
	3rd	28 (5.3)	28 (5.3)	29 (5.5)	29 (5.5)	28 (5.3)	31 (5.8)
	4th	28 (5.1)	21 (3.9)^b^	33 (6.1)	41 (7.5)^a^	28 (5.1)	23 (4.2)
	5th	27 (5.1)	23 (4.3)	27 (5.1)	41 (7.7)^a^	27 (5.1)	28 (5.3)

TC, total cholesterol; HDL-C, high-density lipoprotein cholesterol.

Height-specific cut-off points were calculated from subjects in each quintile, and non–height-specific cut-off points were calculated from all subjects.

The McNemar test was used to compare height-specific and non–height-specific cutoff points.

^a^*P* < 0.01 and ^b^*P* < 0.05, height-specific cut-off point vs non–height-specific cut-off point.

**Table 6.** Prevalence of girls with dyslipidemia by height-specific and non–height-specific cut-off points in each quintile

	Height quintile	Dyslipidemia, number (%)					
	
		Height-specific cut-off point	Non–height-specific cut-off point	Height-specific cut-off point	Non–height-specific cut-off point	Height-specific cut-off point	Non–height-specific cut-off point
		TC		HDL-C		Non–HDL-C	
Age 10	1st	25 (5.1)	29 (5.9)	25 (5.1)	16 (3.3)^a^	26 (5.3)	30 (6.1)
	2nd	24 (5.0)	30 (6.3)^b^	28 (5.9)	16 (3.3)^a^	24 (5.0)	26 (5.4)
	3rd	26 (5.4)	35 (7.3)^a^	26 (5.4)	30 (6.2)	25 (5.2)	37 (7.7)^a^
	4th	26 (5.3)	20 (4.1)^b^	28 (5.7)	36 (7.4)^a^	25 (5.1)	20 (4.1)
	5th	26 (5.5)	9 (1.9)^a^	27 (5.7)	40 (8.4)^a^	25 (5.3)	11 (2.3)^a^

Age 14	1st	25 (5.2)	30 (6.2)	27 (5.6)	23 (4.8)	25 (5.2)	38 (7.9)^a^
	2nd	25 (5.5)	28 (6.1)	30 (6.6)	20 (4.4)^a^	23 (5.0)	26 (5.7)
	3rd	26 (5.5)	18 (3.8)^a^	27 (5.7)	39 (8.3)^a^	25 (5.3)	22 (4.7)
	4th	27 (5.6)	32 (6.6)	28 (5.8)	28 (5.8)	25 (5.2)	25 (5.2)
	5th	25 (5.6)	14 (3.1)^a^	24 (5.4)	36 (8.1)^a^	23 (5.2)	15 (3.4)^a^

TC, total cholesterol; HDL-C, high-density lipoprotein cholesterol.

Height-specific cut-off points were calculated from subjects in each quintile, and non–height-specific cut-off points were calculated from all subjects.

The McNemar test was used to compare height-specific and non–height-specific cutoff points.

^a^*P* < 0.01 and ^b^*P* < 0.05, height-specific cut-off point vs non–height-specific cut-off point.

## DISCUSSION

Using a large population-based sample from school screening in Iwata City, Japan, we attempted to set cut-off points for lipid values stratified by height and age. In an earlier study, TC and HDL-C levels in pubertal children were found to be significantly associated with growth (age and height).^11^ In the present study, there were inverse relationships between height and serum lipid levels. In addition, in the cut-off point for TC level, we observed a difference between the lowest and highest quintiles of height of 16 mg/dL in 10-year-old girls and 12 mg/dL in 14-year-olds boys and girls. The difference in the HDL-C cut-off point was 2 and 4 mg/dL in 10-year-old boys and girls, respectively, and 3 mg/dL in 14-year-olds girls and boys. The difference in the non–HDL-C cut-off point between the first and fifth quintiles of height was 4 and 9 mg/dL in 10-year-old boys and girls, respectively, and 1 and 14 mg/dL in 10-year-old boys and girls, respectively. In addition, there were significant differences in the prevalence of dyslipidemia calculated using height-specific versus non–height-specific cut-off points, especially in the lowest and highest quintiles. These marked differences indicate that it is necessary to consider height in addition to age when evaluating cholesterol levels in pubertal children.

Cholesterol levels are much low in newborns than in adults, and they increase during early childhood.^24^ Serum levels of TC decrease between the ages of 10 and 16 years in boys and 9 and 14 years in girls.^15^ An adult pattern of lipoprotein cholesterol is present by the end of puberty.^15^^,^^18^ Lower HDL-C and low-density lipoprotein cholesterol (LDL-C) levels are associated with increased testosterone in boys and increased estradiol in girls.^25^ It has also been reported that growth hormone therapy causes a decline in LDL-C and HDL-C levels.^26^ Serum lipid levels are influenced by sex hormones and growth hormone.

In our results, the 95th percentiles of TC level (221 mg/dL, 10-year-old boys; 206 mg/dL, 14-year-old boys; 217 mg/dL, 10-year-old girls; 223 mg/dL, 14-year-old girls) were similar to the cut-off points published by the Japan Association of Health Service survey, which was conducted in 19 of the 47 prefectures in Japan. In a Japanese national survey, the 95th percentiles of TC level were 219 and 218 mg/dL in 10-year-old boys and girls, respectively, and 208 and 220 mg/dL in 14-year-old boys and girls.^27^ The fifth percentiles of HDL-C level (45 mg/dL, 10-year-old boys; 43 mg/dL, 14-year-old boys; 44 mg/dL, 10-year-old girls; 46 mg/dL, 14-year-old girls) in the present study were also similar to those used in the national survey. The fifth percentiles of HDL-C levels were 43 and 42 mg/dL in 10-year-old boys and girls, respectively, and 41 and 42 mg/dL in 14-year-old boys and girls in the national survey.^27^ Cholesterol levels from the original Iwata population were therefore consistent with those in the national survey.

A possible limitation of this study is that our data are from only one area in Japan, ie, the subjects were not randomly selected from the whole country. However, mean body height and mean body weight in Year 5 students in 2001 were consistent with those reported in a national survey.^27^ In addition, the 95th percentiles of TC and the 5th percentiles of HDL-C levels were similar to those reported in the same national survey.^27^ The height-specific cut-off points yielded by this study may contribute to school-based screenings conducted in several Japanese cities.^11^^–^^13^ We did not, however, investigate the association between LDL-C level and height. Recently, LDL-C level has replaced TC as the most important risk factor for coronary heart disease in Japan. Further studies are therefore required to investigate the relationship between height and LDL-C and to derive height-specific cut-off points for LDL-C.

Nevertheless, the present study has several strengths, including the large population-based sample (10 152 school children, 98.9% of the target population), the clear description of the study population, the clear and comprehensive description of the annual examinations that were conducted at each school annually, and the fact that this was the first study to report levels of non–HDL-C in Japanese children. In addition, the methods for measuring and analyzing lipid variables are provided, including the intra- and inter-laboratory coefficients of variation (2%–4%).

This study calculated height-specific levels of serum lipids in both 10-year-old and 14-year-old children. The differences between the lowest and highest quintiles of height were marked. Thus, consideration of height is necessary when evaluating cholesterol levels in school-based screening and intervention programs.
